# M. A. Brierre de Boismont on the Employment of Baths in Mania

**Published:** 1848-07-01

**Authors:** A. Brierre de Boismont


### RECENT OBSERVATIONS ON THE PROLONGED EMPLOYMENT OF BATHS AND CONTINUOUS AFFUSIONS OF WATER IN SOME OF THE ACUTE FORMS OF MADNESS, ESPECIALLY MANIA.

(*Read at the Academy of Sciences, at Paris, Feb.* 14, 1848.)

By M. A. Brierre de Boismont.

Acute mania is a form of madness that has long been regarded as a type of its kind, from the rapidity of its course, the variety of its symptoms, and the violence by which it is characterized. It is, however, consoling, in the midst of the formidable symptoms to which it gives rise, to remember that this kind of insanity is more rapidly cured than any other, especially in its most acute stage. All authors have concurred in this opinion; and on examining the duration of the treatment recorded in several statistical reports, it will be found, for instance, that of the 238 cases treated by Esquirol, the greater number were cured at the end of the second or fourth month of treatment. The cures of the 88 cases of mania cited by MM. Aubanel and Thore, were effected between the first and fourth months. On visiting Bedlam and St. Luke's Hospital in London, we learnt that the greater number of the cures were effected between the second and seventh months of treatment. Thus it may generally be established, that in all countries where the insane are treated according to an enlightened and rational method, mania requires commonly from six weeks to two months for its cure. The method that we are about to propose is, in our opinion, preferable to any that has as yet been tried, since it generally requires only one week, and does not exceed a fortnight.

The cases included in our former memoir on the subject of this mode of treatment amounted to 72; of these, 61 were cured by a course of treatment which did not extend beyond a week in the case of three-fourths of the number; whilst for the remainder, it was not prolonged beyond a fortnight.

The means employed were, the prolonged employment of baths and affusions. The patients remained from eight or ten to fifteen hours in covered baths, whilst a current of water was continually poured over their heads. The temperature of these baths was from 82° to 86° Fahrenheit; that of the affusions, 60° Fahrenheit. When the patients left the bath, the heat varied from 64° to 68° Fahrenheit. The therapeutic effects of these baths may easily be understood: among these we may reckon diminution of the circulation and respiration; relaxation of the skin; alleviation of thirst; the introduction of a considerable quantity of water into the economy, (estimated by Falconnet at three pounds per hour;) an abundant discharge of limpid urine; a tendency to sleep; a state of repose, &c.; all of these being actions which essentially contribute to place baths amongst the most decided of our relaxing and calming remedial agents. They take the place of bloodletting, having the great advantage of not removing from the economy a principle which is frequently indispensable. Since the publication of my first memoir, twenty-five new observations have been added to those of which we were previously in possession. The following table gives the results:—

| | | Cured. | Not cured. | Relapsed. | Died. |
|---|---|---|---|---|---|
| Acute mania | 6 | 4 | 1 | 0 | 1 |
| Maniacal excitement | 7 | 7 | 0 | 0 | 0 |
| Puerperal mania | 2 | 2 | 0 | 0 | 0 |
| Intermittent mania | 1 | 0 | 0 | 1 | 0 |
| Delirium tremens | 2 | 2 | 0 | 0 | 0 |
| Acute monomania | 7 | 4 | 0 | 3 | 0 |
| | 25 | 19 | 1 | 4 | 1 |

The most numerous and constant cures have been observed in the case of acute mania and maniacal excitement; and only in one case has the mode of treatment of the prolonged employment of the baths proved unfavourable where the disease was of recent date. This mode of treatment is ineffectual in cases of periodic intermittent mania, in mania beginning with folly, epilepsy, or general paralysis. An hereditary tendency, without being an obstacle to a perfect cure, must be regarded as increasing the difficulty of the case. Chronic mania has only been ameliorated; and this method was not attended by any marked result where acute mania resembled an hysterical form of acute delirium, and where drink was rejected.

In many patients, agitation ceases during the first few hours; in others, it continues during the greater part of the time in which they continue in the bath. In most cases, calmness is established after six or eight hours. The disorder generally recurs some hours afterwards, or in the middle of the night. Immersion determines a rush of blood towards the central parts, which is often appreciable by the redness and tension of the head. To obviate this inconvenience, we have caused a stream of water to flow for hours on the top of the head.

Whatever preference may be given to the prolonged employment of baths, we have not hesitated in associating with them blood-letting, purgatives, and emeto-cathartics, when they have been required by the indications. As many insane patients smell all that is given them, and reject everything having any peculiar taste or smell, we have been accustomed to blend calomel alone, or mixed with an emetic, in their food and drink.

The facts contained in the present memoir justify us in maintaining the conclusions we had already adopted, and which we here subjoin:—

1. The acute forms of insanity, and of mania in particular, may be cured in a period of time varying from one to two weeks.

2. The treatment to be employed consists of baths, (prolonged,) and a continuous stream of water.

3. The relaxation of the circulation and respiration, the introduction of a great quantity of water into the system, and the gradual and general cooling, demonstrate that these baths have an action essentially calming and sedative.

4. The duration of the baths should in general be from ten to twelve hours; they may be prolonged to fifteen or eighteen hours.

5. The stream of water should be kept up during all the time the bath is applied; it may be suspended when the patient is tranquil.

6. The bath ought to be given at the temperature of from 28° to 30° C. (82° to 86° F.), and the stream at 15° C. (43° F.)

7. Of the forms of mania, that which yields most to the action of baths and irrigations is acute mania; next follow simple acute delirium, delirium tremens, the madness of puerperal women, and monomania with acute symptoms; but in most of these forms the cures are neither so rapid nor so permanent as in acute mania.

8. The period of convalescence ought to be strictly attended to, inasmuch as relapses are by no means of rare occurrence, whenever the individuals are exposed to the influence of the same causes which have produced the disease.

9. When acute mania borders on acute delirium, with an ataxic form, and rejection of drinks, the treatment is ineffectual.

10. Chronic mania, or prolonged acute mania, with agitations, have been improved, but not cured by this treatment.

11. From the facts contained in the two memoirs, we may assert that the cures of acute forms of insanity, and of mania in particular, are more numerous and expeditious, by means of protracted baths and irrigations, than those obtained by other treatment; since whilst by the latter the medium duration of treatment is about six weeks, it is no more than eight days when baths and irrigations are employed.

12. These baths, &c., appear to us to promise great benefit in cases of hysterical affections, and in many other nervous diseases accompanied by excitement.

13. The prolonged baths have no inconvenience; the fatigue they may induce is easily dissipated. They do not deprive the system of any important principle, and they do not leave behind any of those confirmed debilities so often observed after abundant venesections, and the fatal termination of which has more than once been dementia.

14. The employment of protracted baths is no novelty in science; but till this period, this mode of employment which can be tried everywhere has not been applied to the treatment of special cases. Their union with the stream of cold water, however, constitutes a new method.

